# The prefrontal cortex: from monkey to man

**DOI:** 10.1093/brain/awad389

**Published:** 2023-11-16

**Authors:** Richard Levy

**Affiliations:** AP–HP, Groupe Hospitalier Pitié-Salpêtrière, Department of Neurology, Sorbonne Université, Institute of Memory and Alzheimer’s Disease, 75013 Paris, France; Sorbonne Université, INSERM U1127, CNRS 7225, Paris Brain Institute- ICM, 75013 Paris, France

**Keywords:** cognition, behaviour, human, primate, frontal lobes

## Abstract

The prefrontal cortex is so important to human beings that, if deprived of it, our behaviour is reduced to action-reactions and automatisms, with no ability to make deliberate decisions. Why does the prefrontal cortex hold such importance in humans? In answer, this review draws on the proximity between humans and other primates, which enables us, through comparative anatomical-functional analysis, to understand the cognitive functions we have in common and specify those that distinguish humans from their closest cousins.

First, a focus on the lateral region of the prefrontal cortex illustrates the existence of a continuum between rhesus monkeys (the most studied primates in neuroscience) and humans for most of the major cognitive functions in which this region of the brain plays a central role. This continuum involves the presence of elementary mental operations in the rhesus monkey (e.g. working memory or response inhibition) that are constitutive of ‘macro-functions’ such as planning, problem-solving and even language production.

Second, the human prefrontal cortex has developed dramatically compared to that of other primates. This increase seems to concern the most anterior part (the frontopolar cortex). In humans, the development of the most anterior prefrontal cortex is associated with three major and interrelated cognitive changes: (i) a greater working memory capacity, allowing for greater integration of past experiences and prospective futures; (ii) a greater capacity to link discontinuous or distant data, whether temporal or semantic; and (iii) a greater capacity for abstraction, allowing humans to classify knowledge in different ways, to engage in analogical reasoning or to acquire abstract values that give rise to our beliefs and morals. Together, these new skills enable us, among other things, to develop highly sophisticated social interactions based on language, enabling us to conceive beliefs and moral judgements and to conceptualize, create and extend our vision of our environment beyond what we can physically grasp. Finally, a model of the transition of prefrontal functions between humans and non-human primates concludes this review.

## Teaser

Although there is a continuum between human cognition and that of apes, Richard Levy argues that it is the development of our prefrontal cortex that distinguishes us from the other primates by providing us with the capacity for abstraction and the ability to extract ourselves from the present by mentally travelling into the past and future.

## Introduction

### How are we so similar to other primates and yet so different?

Seven to twelve million years ago, our oldest ancestors gave birth to two separate branches of *Hominidae* leading to modern humans (*Homo sapiens*) and the great apes, including chimpanzees, our closest cousins with whom we share 98.8% of our genes.^1,2^ Behaviourally and cognitively, modern humans and chimpanzees (along with other great apes) share many common traits such as the ability to make tools (e.g. they use wooden sticks to catch termites),^3^ initiate complex social interactions (e.g. going to war clan against clan)^4^ or demonstrate some form of self-consciousness (e.g. recognizing themselves in a mirror).^5^ However, it is not only primates that know how to use tools (e.g. crows know how to do this)^6^ or have self-awareness (e.g. elephants can also recognize themselves in a mirror).^7^ In consequence, whatever the cognitive abilities present in non-human primates (including chimpanzees), they are clearly different from those of humans (for a review, see Read *et al*.^8^). The level of civilization that humans have achieved over time tends to demonstrate that humans are far more distant from other great apes than great apes are from old-world monkeys such as the rhesus monkey and, more globally, from other mammals. Indeed, while all living species, including non-human primates, follow the established order of nature, doing their best to adapt and survive, we as humans seek to distance ourselves from this natural order through creativity and progress, repelling disease, improving our comfort and travelling. Why do humans perceive that another order is possible? Could this be the result of a considerable expansion of our imaginative capacities? Imagination (i.e. the faculty or action of forming new ideas, images or concepts of external objects not present to our senses) requires interesting abilities such as maintaining and manipulating mental representations (i.e. working memory); expending temporal space to forecast ourselves in the future; linking distant semantic knowledge to think away from pre-established associations (i.e. creativity); having access to analogical reasoning (e.g. ‘the earth is to the sun what the electron is to the nucleus’); and abstract thinking (e.g. ‘in what way are oranges and bananas alike despite their physical differences?’). All of these cognitive abilities that seem to reside in the human species extend our imaginative representation of the future to a degree that far exceeds that of any other animal species. Interestingly, in humans, all of these cognitive skills require the full integrity of the prefrontal cortex.^9,10^ Inversely, diseases damaging large portions of the prefrontal cortex render us apathetic and impulsive, keep us in the present, incapable of abstraction, subjugate us into automatic thoughts or make us respond immediately to the flow of our percepts or push us to satisfy our primary needs rather than control them and develop behaviour based on mental deliberation.^9,10^ How do we explain, from these observations, the particularity of human cognition and its relationship to the human frontal lobes? How do we see, in an evolutionary vision, the role in humans of the prefrontal cortex in the transition(s) from the state of being an animal, fully integrated into the order of things, to the condition of being civilized, presenting consciousness, abstract thinking, language, reasoning and other higher cognitive functions? This pushes forward a subsequent ontological question: is there a gap or a continuum between human and non-human primates in terms of higher cognitive functions and underlying brain structures that serve these functions? This review attempts to provide some answers to these questions.

## The general principles of prefrontal functions in humans

The anterior part of the human brain received little attention in science and medicine until the midst of the 20th century, when it was observed that frontal lobotomies provided treatment for agitated psychotic patients in such a way that after this procedure, patients were calm and peaceful (or should we say ‘apathetic’?).^11^ Since then, the understanding of the functional role of the prefrontal cortex has started to emerge.^12^ The most powerful and influential concept was proposed by Alexandre Romanovich Luria, who postulated that the prefrontal cortex is the key structure in elaborating and controlling ‘conscious’, ‘voluntary’, ‘willed’, ‘non-automatic’ and ‘goal-directed’ actions.^13^ To date, this general concept remains the central underlying idea on which the main current neuroscience models regarding the functional anatomy of the prefrontal cortex are based.^14-25^ Of course, what makes this concept appealing is the idea that a particular evolution of the anterior part of the brain in *H. sapiens* is associated with humanity’s ability to escape a repetitive, uncontrolled fate and create its own history.

In the later decades of the 20th century, scientific discoveries were made supporting the idea that the prefrontal cortex controls willed and conscious actions. For example, in non-human primates, it was found that one important particularity of the prefrontal cortex is to maintain and manipulate mental representations of the past (our past experiences, our values and beliefs), the present (the ongoing percept) and the future (the path to follow and that to avoid, the plan of actions to compose or the perspective of the consequences of our choice).^14,26^ These representations exist in a common mental space favouring deliberation and decision-making and allow us to overcome the reflexive cycle between perception and action. Without this mental space of representations, behaviour would consist only of automatic responses to the immediate flow of internal and external percepts (visceral, emotional, visual, auditory, tactile, etc.). Interestingly, this general concept is supported by the clinical observations of the consequence of prefrontal damage. French neurologist François Lhermitte has described the ‘environmental dependency phenomena’ in patients with severe prefrontal damage; these patients could not resist the attraction or pressure their environment exerted on them.^27,28^ For instance, when an examiner sat in front of such a patient, without looking the patient in the eye, and put his hands in the patient’s hands without giving any instruction, the patient spontaneously grasped them and could not stop or control this action. When the patient was asked not to grasp the examiner’s hands and then questioned about what he/she was supposed to do, the patient would answer: ‘I know I should not take your hands, but I cannot help it’. It is also possible to observe imitation behaviour (a patient may imitate different gestures like clapping hands), utilization behaviour (manipulation of objects that the examiner put on a table in front of patients) or collectionism (patients may be unable to prevent themselves from collecting items, sometimes in a well-planned manner).^27,29^ It can be concluded that without the prefrontal cortex, only archaic, impulsive, automatic, overlearned, non-flexible and invariant responses would be possible. To support this increased capacity for mental deliberation in humans, structural and functional differences may be observed between human and non-human primate prefrontal cortices.

Prior to discussing the anatomical and functional similarities and differences between human and non-human primates, it is necessary to define the prefrontal cortex structurally in non-human primates. What is the prefrontal cortex? This seemingly easy-to-answer question is, in fact, quite difficult to resolve.

## What is the prefrontal cortex in humans? —a more complex issue than it appears

Contrary to what may seem obvious, the human prefrontal cortex cannot be defined solely by the fact that it is located at the front of the brain. Indeed, while the most anterior part of the brain, the Regio frontalis,^30^ is relatively well circumscribed macroscopically on its lateral surface—it lies in front of the primary motor and premotor cortices—its caudal boundaries in the ventral and medial regions are less clear. Alternatively, one might prefer to define the prefrontal cortex on the basis of a cytoarchitectonic criterion (i.e. the qualitative and quantitative distribution of cells in cortical layers). According to this criterion, Brodmann defines the prefrontal cortex as an isocortex (six-layer cortex) in which the fourth layer, composed of granular cells, is particularly developed.^30^ This region is therefore called the ‘granular’ cortex. However, a definition using only this cytoarchitectonic criterion would not describe the prefrontal cortex in its entirety. It would exclude the posterior portions of the orbital cortex and the ventromedial regions, which are agranular.^31^ Alternatively, a hodological criterion (i.e. its specific anatomical connectivity with other brain structures), based on retrograde degeneration following cortical lesions, defines the prefrontal cortex according to its inputs from the mediodorsal thalamic nuclei.^32^ The hodological criterion allows the extension of the prefrontal cortex to the posterior ‘agranular’ orbital and ventromedial regions. However, mediodorsal thalamic nuclei also project to the premotor, parietal and temporal cortices and the posterior insula, or the parahippocampal gyrus, although these inputs are smaller than in the frontal regions.^33-39^ Therefore, taken in isolation, this criterion is too inclusive. Finally, the most acceptable anatomical definition is based on the combination of cytoarchitecture and connectivity, allowing us to consider the human prefrontal cortex as being formed mostly of a granular isocortex in the dorsolateral, ventrolateral, anterior orbital and ventromedial areas of the frontal cortex, excluding premotor, temporal, parietal and insular cortices. However, because none of these anatomical criteria alone provides a non-disputable definition of the prefrontal cortex and the fact that pure anatomical criteria are blind to brain functions, it is likely that in humans a better definition would be based on functional neuroanatomy.^40-43^

## Subdivisions of the human prefrontal cortex based on functional neuroanatomy

**Figure 1:**
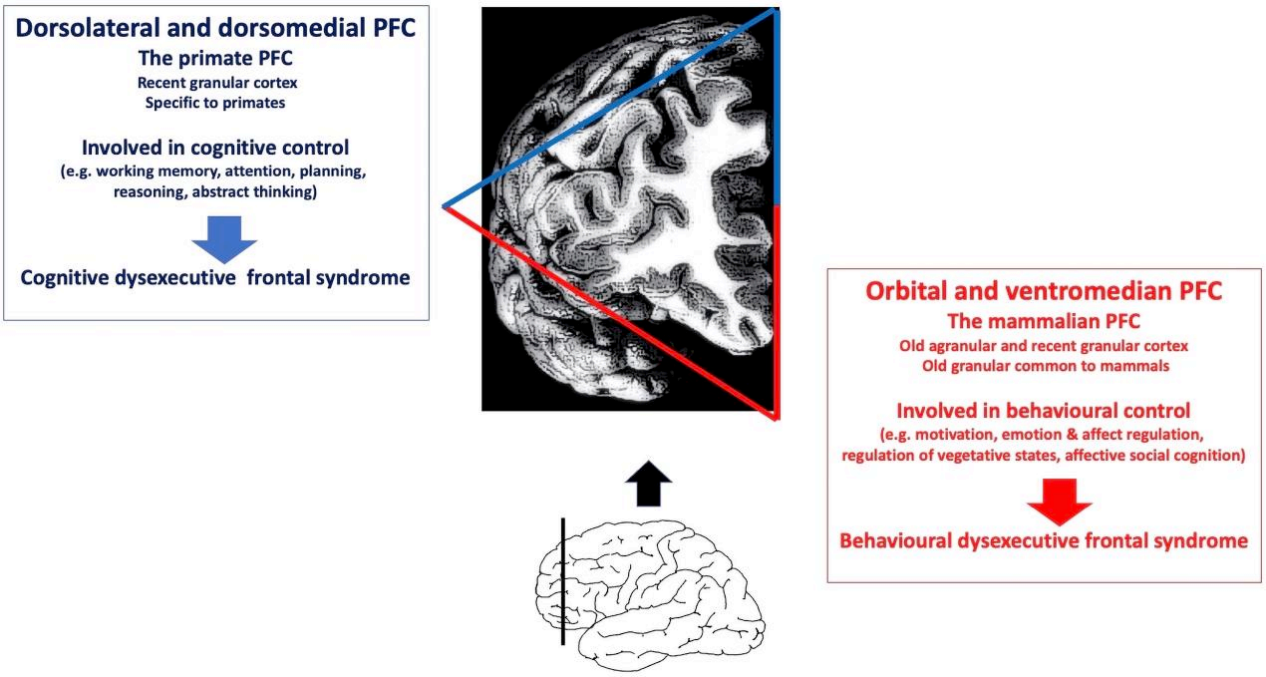
**The two canonical prefrontal regions and syndromes in humans**. PFC = prefrontal cortex.

In terms of anatomical and functional architecture, the prefrontal cortex cannot be considered homogeneous. Although the precise nature of the subdivisions within the prefrontal cortex is still debated, there is no doubt that it can be subdivided into many subregions according to its functional and connectivity properties.^40,44-47^ One very simple, meaningful subdivision in terms of functions, phylogeny and anatomical properties, as well as being clinically efficient, is the separation of the prefrontal cortex into two canonical regions (Fig. 1): (i) the orbital and ventromedial; and (ii) the lateral (dorso- and ventrolateral) and dorsomedian, prefrontal cortex.

The orbital and ventromedial prefrontal cortex is connected mainly to vegetative systems such as the hypothalamus, to anatomical emotion-processing systems such as the amygdalar nuclei and to structures involved in reward or punishment processing such as the ventral striatum.^31,48-56^ It is involved in the control of vegetative functions, emotions and affects, valuation processing, and decoding contexts for decision-making or social cognition, notably through the affective component of empathy^57-68^ (see Box 1 for additional information).

Box 1 **Relevant subject areas that are not included in this review**

**Regions of the prefrontal cortex not discussed in this review:**

- The orbitofrontal cortex, which has a role in social cognition, the regulation of emotions, affectations and vegetative states.^69-72^
- The ventromedial prefrontal cortex, which has a role in reward processing and valuation.^66,70,73^
- The anterior cingulate cortex, which has a role in conflict/error monitoring, awareness and reward-based learning.^68,71,74^

**Why are these regions not discussed in this review?**

There is no doubt that these three other large regions of the prefrontal cortex play crucial roles in goal-directed cognition. The choice has been made to illustrate the similarities and differences between the prefrontal cortex of humans and that of non-human primates using the example of the lateral prefrontal cortex because it best characterizes the primate prefrontal cortex. The other three prefrontal regions contain a mixture of old (not specific to primates) and recent (specific to primates) subregions.

It is important to note that these three other prefrontal areas exhibit major differences from the lateral prefrontal cortex in terms of functional specialization and anatomical structures (connectivity and/or cytoarchitecture and/or phylogeny and/or ontogeny). Therefore, the different models of organization of the lateral prefrontal cortex presented in this review do not describe the prefrontal cortex globally, although an attempt to do so has been published by Kouneiher *et al.*^75^

**Additional functions that involve the prefrontal cortex:**

- Attention.^76^
- Arousal and awareness/consciousness.^77-79^

**Why are these functions not discussed in this review?**

Attention, although not a single brain function, overlaps strongly with working memory at all levels of approach: conceptual, behavioural, psychological or cognitive mechanistic, neural network and cellular levels (for a recent review, see Bahmani *et al.*^76^). Therefore, the discussion about the role of the lateral prefrontal cortex in working memory could to a large extent be applied to attention.

Whatever the most recent theories underlying the concept of consciousness/awareness (e.g. the Higher Order or Global Workspace theories), they all rely on a largely distributed network of brain regions in which the lateral prefrontal cortex plays an important role.^80,81^ The way the involvement of the lateral prefrontal cortex in consciousness/awareness is usually described fully overlaps with what is required to achieve a goal-directed behaviour^77,78^ as discussed in this review.

Recently, besides the large body of studies focused on the quality of conscious experience, emerging evidence also pleads for a role of the prefrontal cortex in regulating of the level of arousal.^79^ However, the specific subregions associated with the primate prefrontal cortex remain to be established.

In contrast, the lateral and dorsomedian prefrontal cortex, which is entirely granular, has recently emerged as a primate apparatum.^25,31^ It is connected mainly to multimodal associative regions (i.e. areas that manage information from multiple sense modalities) related to the most integrated sensory processing within the temporal and parietal cortices.^26,82^ It represents a critical structure in the brain circuits of attention, the manipulation and updating of information in working memory and executive cognitive functions such as abstraction, reasoning or planning^14,17,18,26,46,83-91^ (see Box 1 for additional information).

According to this subdivision, two main frontal syndromes, ‘orbital/ventromedial’ and ‘lateral’ prefrontal syndromes can be distinguished. Orbital/ventromedial prefrontal syndrome is representative of pathologies such as the behavioural variant of frontotemporal dementia, cerebral haemorrhage due to the rupture of an aneurysm of the posterior communicant, or meningiomas developed at the expense of the greater wing of the sphenoid bone. It is characterized by vegetative disorders (e.g. disturbances in urinary control or gluttony), disorders in affect and emotion regulation (e.g. emotional blunting or lability), motivational processes (e.g. apathy or inability to evaluate the future consequences of our actions) and disturbances in social interactions (e.g. social disinhibition or loss of empathy with egocentric withdrawal).^9,10,92-98^ Lateral prefrontal syndrome has many neurological and psychiatric causes (from gliomas to ischaemic strokes as well as depression or schizophrenia). It is characterized by classical ‘cognitive dysexecutive syndrome’, including working memory impairments, attention disorders, difficulties in cognitive flexibility, cognitive inhibition, abstraction or planning.^44,87,99-108^

We must next ask, to what extent is the anatomical-functional organization of the human prefrontal cortex found in the non-human primate?

## Anatomical similarities and differences between the prefrontal cortex of human and non-human primates

**Figure 2:**
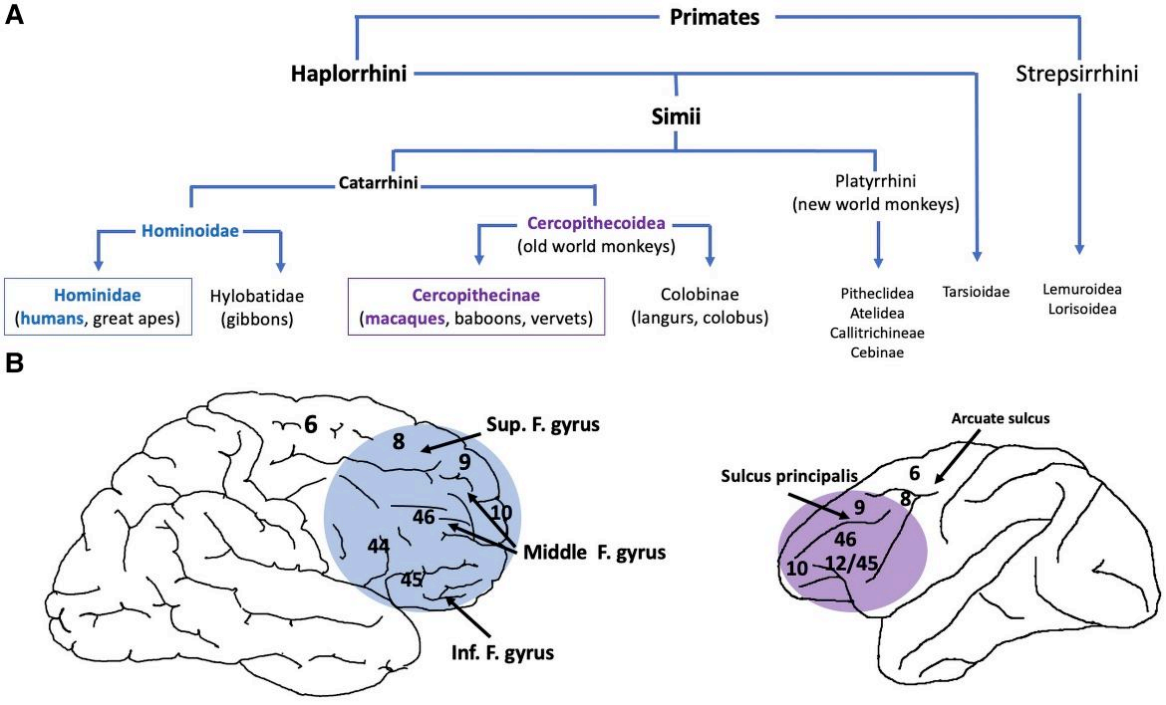
**Phylogeny and comparative anatomy in primates.** (**A**) Phylogeny of the order of the primates. (**B**) Comparative macroscopic anatomy of the lateral prefrontal cortex in humans and the rhesus monkey. Numbers refer to the cytoarchitectonic maps compiled by Brodmann for humans and Walker for the rhesus monkey.

Which primate(s) is/are compared to humans? The vast majority of studies on the comparative human/non-human anatomy and functions of the prefrontal cortex have focused on macaques, in particular, the rhesus monkey (*Macaca mulatta*), and to a lesser extent, the chimpanzee (*Pan troglotydes*). These species belong to the Catarrhini branch from which the Hominidae (humans and great apes) and Cercopithecinae (baboons, macaques and vervets) emerged^31,109^ (Fig. 2A). It is therefore important to preface that this focus on two species of primates—the rhesus monkey and the chimpanzee—which are certainly quite close to humans on the phylogenetic tree, should prevent us from over-generalizing.

### Similarities

The first important similarity between the prefrontal cortex of humans and non-human primates, particularly within the Catarrhini (including humans and the rhesus monkey), is its cytoarchitecture. The dorsolateral, ventrolateral, anterior mesial, anterior ventral and orbital regions as well as the rostral surface of the prefrontal cortex are considered a ‘granular isocortex’.^31^ In humans, the lateral (dorsolateral, ventrolateral and lateral rostral) prefrontal cortex) is represented by Brodmann areas (BA) 8, 9, 46, 44/45, 47 and lateral 10. In the rhesus monkey, it is represented by Walker’s areas 8, 9, 10, 12/45, 46, 47 and 10. This isocortex seems to differentiate the primate prefrontal cortex from the anterior portion of the brain of other mammal species in which the cortical surface is agranular. Nevertheless, it is important to note that the posterior ventromedial (BA 22, 25, 32) and posterior part of the orbital (BA 13) prefrontal cortex are agranular cortices.^31,48^

A second similarity between the prefrontal cortices of humans and non-human primates (including great apes and rhesus monkeys) is the presence in layer Vb of von Economo neurons (and fork cells). These neurons are located in mesial BA9 and BA10 in the insula and in the anterior cingulate cortex. They are thought to be the prefrontal nodes of the salience network, and they are particularly vulnerable to the degenerative process that occurs in frontotemporal lobar degeneration, particularly in cases associated with *C9orf72* hexanucleotide gene expansion. However, one should note that von Economo neurons are not specific to the primate brain and have also been found in the brains of cetaceans, elephants and manatees.^110-121^

Third, in primates, the prefrontal cortex and basal ganglia are organized into several relatively parallel loops connecting different regions of the frontal lobes to specific portions of the striatum, the internal pallidum/pars reticulata of the substantia nigra (the non-dopaminergic part of the substantia nigra), and certain nuclei of the thalamus, and then back to the cortex from where the fibres originated.^122^ These connections follow the same general pattern of organization (cortex-striatum-pallidum or substantia nigra pars reticulata-thalamus-cortex) but differ from others in their cortical and subcortical territories.^123-130^ Therefore, the dorsolateral prefrontal cortex projects to the dorsolateral part of the basal ganglia. The more ventral prefrontal cortex (ventromedial) projects to the more ventral portion of the basal ganglia. These prefrontal basal ganglia loops form relatively separate functional systems, although recent studies show that some degrees of convergence occurs through basal ganglia funnelling, allowing for the integration of diverse cortical areas.^130,131^ Such a model of organization is also observed in humans, thanks to *in vivo* connectivity studies using 3D-MRI diffusion tensor axonal tracking.^132-134^

As a consequence, in both humans and the rhesus monkey, lesions in any part of these loops can induce cognitive or behavioural changes that resemble those of direct lesions in the prefrontal regions to which these nodes are connected, and these changes are expressed sometimes more severely than direct lesions of the prefrontal cortex.^135-145^ Therefore, lesions to the dorsal head of the caudate nucleus (connected to the dorsolateral prefrontal cortex) produce impairments in working memory, cognitive dysexecutive syndrome and apathy, whereas lesions of the ventral striatum (connected to the ventromedial prefrontal cortex) result in impaired valuation processing.

Lastly, the rostral and lateral part of the prefrontal cortex (BA10, also called the frontopolar cortex) is particularly developed in non-human primates. As we will see later, this region of the prefrontal cortex plays a central role in our ability to abstract, reason and be creative. It is therefore not surprising that the frontopolar cortex is much more developed in humans than in all other mammalian species, including the great apes^146-148^ (Fig. 2B).

### Differences

The most striking difference between human and non-human primates regarding the prefrontal cortex is its size. Indeed, of all mammal species, it is in humans that the prefrontal cortex is the most developed in proportion to the rest of the brain. According to Brodmann, the prefrontal cortex represents up to 29% of the surface of the human cerebral cortex, while it represents 17% and 11.5% of that in the chimpanzee and the rhesus monkey, respectively.^30^ A more recent analysis indicates that the proportions of grey and white matter in the human prefrontal cortex are 1.9–2.4-fold that of a rhesus monkey and about 1.2–1.7-fold that of a chimpanzee.^149^ In humans, this increase in size is hyper-allometric and spectacular (in biology, allometry is the fact that organs, tissues or processes grow at different rates). Indeed, Smaers *et al.*^150^ have shown that when comparing the size of the prefrontal cortex to that of the frontal motor areas or the primary visual cortex across various species of primates from the smallest Lemurians to the great apes, the increase in the surface of the prefrontal cortex parallels that of the other cortices, except in great apes, where it increases much more than the other cortices; and in *H.sapiens* the increase is outstanding. This increase concerns both the white and grey matter.^149,151^ However, within the grey matter, the proportion of prefrontal neurons (8% of cortical neurons) remains lower than the expected number of neurons, given the relative size of the prefrontal cortex.^152^ What increases the most is the neuropil (i.e. the number of dendrites and axons)^153^ and the glial density (i.e. the number of layer I astrocytes).^154^ Therefore, the increase in size seen in humans is likely to be due to the increased intrinsic (i.e. local) and extrinsic (i.e. with other brain structures/cortices) connectivity of the prefrontal cortex. Following this line of reasoning, using functional MRI (fMRI) resting state sequences to assess functional connectivity in the rhesus monkey and in humans, Mantini *et al.*^155^ provided evidence for two novel lateralized frontoparietal networks in humans that are not found in monkeys.

This increase in prefrontal cortex size is translated into an increase in gyration in humans. For instance, on the lateral surface of the human prefrontal cortex, three gyri (the superior, middle and inferior frontal gyri) are observed, along with several sulci according to the most recent proposed nomenclature (the superior, middle, posterior middle, horizontal rami of the intermediate and dorsal para-intermediate frontal sulci),^148^ while in the rhesus monkey, there is only one sulcus—the sulcus principalis—dividing the lateral surface into the dorsolateral and ventrolateral regions. However, in the rhesus monkey, the region encompassing the sulcus principalis and its banks (called the mid-dorsolateral region) is quite large and may represent up to 50% of the lateral prefrontal cortex, because most of the cortex of this region develops in the depths of the fold of the sulcus principalis^26^ (Fig. 2B). Nevertheless, despite these major differences, it is possible to establish anatomical analogies at the macroscopic and network levels between the prefrontal cortex of humans and that of rhesus monkeys as demonstrated in recent years using modern neuroanatomical methods.^43,148,155-157^

### Summary

Taken together, the anatomical data show that non-human primates and humans have in common: (i) the development of a granular prefrontal cortex that differentiates them from other mammals that have an agranular forebrain cortex (which in primates may correspond to the posterior ventromedial and orbital cortices); (ii) the presence of von Economo neurons (although this is not completely specific to primates); and (iii) similar prefronto-subcortico-prefrontal connectivity. In contrast, the human prefrontal cortex has developed dramatically compared with that of other primates, including the great apes. This development seems macroscopically to concern the most anterior part, the frontopolar cortex (whereas cross-species similarities can be established for the rest of the prefrontal cortex), and has allowed for a significant increase in the number of neurons, an extraordinary development in connectivity. This leads us to the following question: how can a quantitative increase in size (and connectivity) lead to a qualitative functional difference in behaviour?

## The lateral prefrontal cortex: a continuum rather than a gap between humans and non-human primates

Because the granular lateral prefrontal cortex in its entirety seems to be specific to primates (as opposed to the orbital and ventromedial prefrontal cortex, which is in part granular but also agranular in its most posterior aspect), we will take this as a model for describing the anatomical-functional similarities and differences between humans and non-human primates.

From an anatomical and functional point of view, the lateral prefrontal cortex can be divided into two subregions, the dorsolateral (corresponding to BA 9/46 in humans and Walker areas 9/46 in the rhesus monkey) and the ventrolateral (corresponding mostly to BA 44/45/47 in humans and Walker areas 12/47 in the rhesus monkey). Despite apparent differences in terms of gyri and sulci, there is relative homology between the lateral prefrontal cortex of the rhesus monkey and that of humans, allowing a ‘gyrus-to-gyrus’ functional comparison in cognition and behaviour, with the notable exception of the lateral frontopolar regions of humans, which have no true equivalent in the rhesus monkey.^148^ How can this relative anatomical homology between humans and the rhesus monkey, at least for most of the lateral prefrontal cortex, be translated into similarities and differences in terms of function?

### The critical role of the dorsolateral prefrontal cortex in planning and working memory

**Figure 3:**
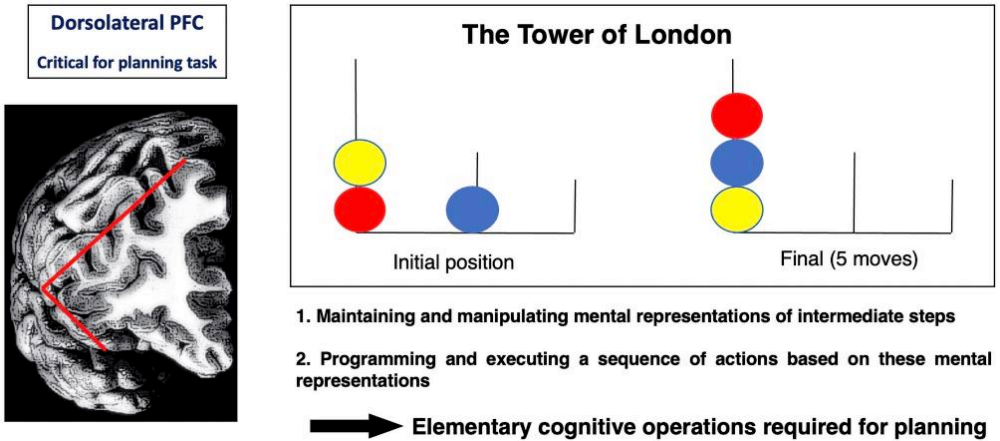
**The ‘Tower of London’, a prototypical planning task.** *Left*: The lateral prefrontal cortex (PFC) is central to planning tasks. *Right*: An example of the Tower of London (ToL) task. The ToL task consists of transferring three coloured disks between three vertical rods, from an initial position to a pre-specified goal arrangement. Here, the solution comes in five moves. Solving the ToL problem within a limited number of moves requires planning the sequence of actions before starting to move the discs. It relies on more elementary cognitive operations such as those proposed at the *bottom*.

Let us consider planning abilities, the proper functioning of which is generally associated with the structural and functional integrity of the dorsolateral prefrontal cortex. Indeed, planning tasks activate the dorsolateral prefrontal cortex constantly (PET/fMRI), and damage to the dorsolateral prefrontal cortex affects planning.^13,84,89,90,100,103,158-161^ A typical task used to study planning is the ‘Tower of London’ task (Fig. 3).^100^ This is a problem-solving task in which one must find the path from an initial position to a final position in a given number of moves. This planning/problem-solving task can be considered a macro-function, as it requires the performance of more elementary operations. Many of these operations are parts of the working memory system, i.e. a set of operations allowing for the maintenance and manipulation of mental representations to guide future actions by ‘representation of stimuli rather than by the stimuli themselves’.^26,162^ Many human activities involving higher cognitive functions, including focused attention, language comprehension and production, reasoning, abstract thinking or planning rely on the full integrity of working memory processes.^162^ Working memory can therefore be considered as a set of elementary operations required for higher cognitive functions. It is possible to break planning tasks down into more elementary operations and then study each of these in isolation (e.g. maintenance, manipulation of mental representations, inhibition of prepotent responses, preparation of forthcoming responses) in monkeys by observing their behaviours in dedicated tasks combined with pre- and post-ablation/inactivation of discrete prefrontal regions or single cell recordings. It is then possible to determine what elementary operation(s) is/are present in planning tasks and critically involve the dorsolateral prefrontal cortex.

### The delay neurons: a major discovery

‘Delayed Response’ tasks have been a primary instrument for assessing working memory capacity in the non-human primate.^163,164^ Their key feature is that they require monkeys to maintain a mental representation during a delay period and then use this representation to guide the response choice at the end of the delay.^163^ In Delayed Response tasks, the monkey must find a hidden reward. During the first phase, a monkey sees a tray with two wells (left and right). In one of the wells, the experimenter places a reward and then covers the two wells with two identical plates. Immediately afterwards, an opaque screen is placed between the animal and the tray for a period of a few seconds (usually 5–10 s), the ‘delay’. After the delay, the screen is raised, and the monkey must move the plate to reveal the reward-containing well. From one trial to the next, the position of the reward is distributed in a pseudo-random order. During the delay, the monkey has no access to visual information that might indicate the correct response. This forces the animal to maintain a ‘trace’ (or internal representation) of the relevant information. It is precisely this active maintenance of an internal representation and the use of this ‘mental image’ of the environment that is at the heart of the behaviour in this task.

Monkeys with dorsolateral prefrontal lesions cannot perform the delayed response tasks correctly.^164^ The deficit is so severe that the response choices correspond to the level of performance dictated by chance. If the delay is removed, frontal lesions no longer produce a deficit. These data suggest that the observed deficit is related to a difficulty in maintaining and/or manipulating and/or using mental representations, introducing the idea that the dorsolateral prefrontal cortex plays a critical role in the storage of mental representations.^165-168^

**Figure 4:**
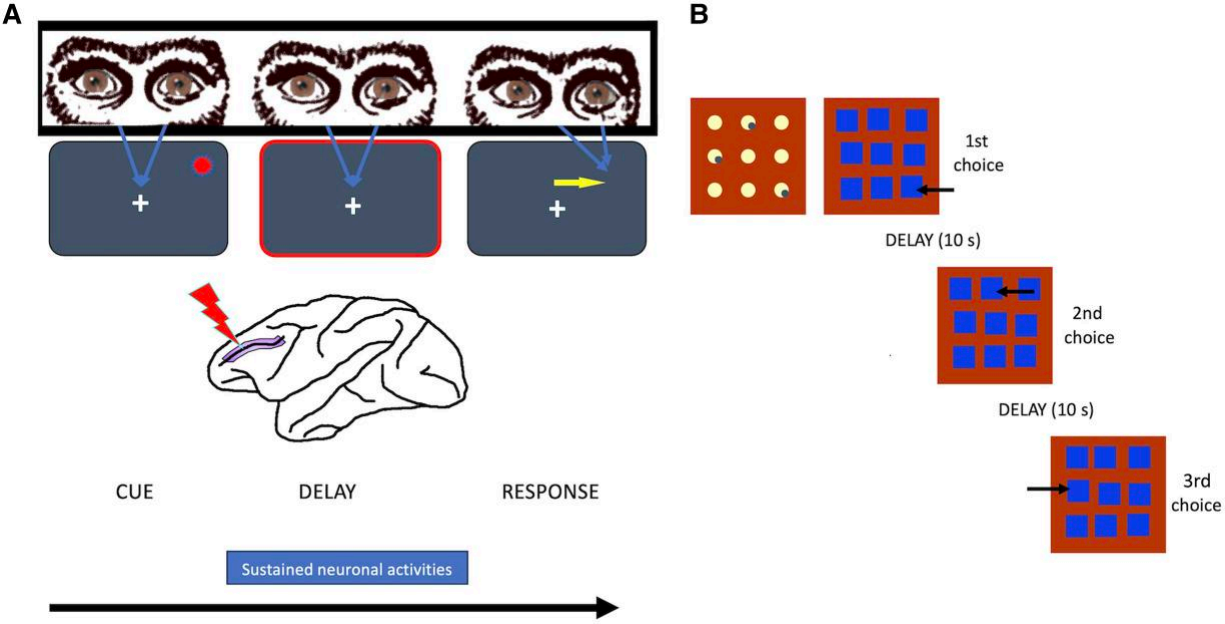
**Examples of working memory and planning tasks performed by the rhesus monkey.** (**A**) The oculomotor delayed response paradigms and their neuronal correlates. In this task, the monkey stares at a central point on a screen. A bright spot then appears in the periphery (at 13°, laterally). The animal must keep its gaze on the central fixation point. The lateral point of light disappears for a few seconds (this is the ‘delay’). At a sound signal, the monkey must make an ocular saccade towards the position where the light point was (it is not present at the time of the ‘response’). At the time of the response, the eye saccade is directed towards the presumed position of the visuo-spatial stimulation, but this stimulus is no longer present. Therefore, action is guided upon the mental trace of the stimulus. (**B**) The spatial Self-Ordering task. In the Self-Ordering task, the monkey sees a tray with nine wells, three of which contain rewards. The overall objective of the task is to find the three rewards in succession, without ever returning to a well already visited. The monkey starts by making a first choice after one, then a second and a third, each choice being separated by a 10-s ‘delay’, during which the visited well is again covered by a plate. If the animal returns to a visited well or goes to an unrewarded well, the trial stops and the animal does not get the maximum possible reward. The task consists of 30 trials. In each trial, the position of the rewards is reconfigured. The task is considered complete when the monkey is able to perform more than 90% of the trials correctly for five consecutive days. All monkeys were able to achieve this goal, demonstrating planning ability.

One of the most illuminating discoveries in behavioural neuroscience was the identification of a significant proportion of neurons in the lateral prefrontal cortex that, during Delayed Response tasks, maintain their activity during the delay and up until the ‘response’.^169,170^ This observation was made possible by placing an electrode in close proximity to a targeted neuron within the area of interest (this is an extracellular single cell recording) and recording its activity while a monkey performed Delayed Response tasks. This was the first evidence of a neuronal correlate of working memory. Using these tasks, and many variations of them such as the ‘Oculomotor Delayed Response’ or the ‘Oculomotor Anti-saccade Delayed’ paradigms (Fig. 4A), it was shown that this sustained activity during the delay could be related to different aspects of the task performance. These include: the maintenance of internal representations of external stimuli in the. short-term memory^171,172^; the response preparation^171^; the inhibition of immediate-prepotent impulsive responses^171^; the sequential order^173,174^; decision-making^175^; the implementation of the context and the rule format^176,177^; and the reward value of the context.^178^ Some prefrontal neurons can respond to multiple relevant aspects of the task such as sensory cues, the task rule and the motor response.^176,179^ This mixed selectivity may be central to the flexibility that characterizes prefrontal functions.^180,181^

The latest research shows that by focusing too much on the sustained activity of neurons in the prefrontal cortex during working memory tasks, the involvement of these neurons in working memory is not fully understood.^182-187^ First, the absence of continuous maintenance of activity during the delay period does not prevent excellent performance in working memory.^184^ Second, it has been shown recently that neuronal activities linked to working memory circulate and oscillate spatially in a network composed of a large number of prefrontal neurons, which favours the division of activities linked to working memory.^187^ Third, prefrontal neurons involved in the short-term maintenance of information in working memory show rapid changes in stimulus selectivity.^186^ This could be considered a putative mechanism for rapid set-shifting abilities, a classical prefrontal function. Therefore, stable active maintenance of the mnemonic trace in working memory in one single prefrontal neuron may not be as critical as one may expect.^113,181,185^ Using approaches such as computational modelling, it has been proposed that the working memory trace can be maintained by ‘dynamic coding’ processing, i.e. a temporal variability in neuronal activity, particularly in a discontinuous but effective manner. For example, this could be achieved by means of an oscillatory flow, enabling numerous neurons to share, at different times during task execution, functional activities related to working memory tasks.^187^ Some data indicate that this is made possible through short-term changes in the synaptic plasticity and efficacy, suggesting that the mnemonic trace can easily be reactivated at a lower energetic cost than active maintenance.^182,183,185^ Taken together, these data indicate that the efficacy of working memory processing does not necessarily rely on sustained activity at the level of a single neuron but rather on dynamic and executive types of processing in which prefrontal neurons interact with each other within a large network.

Overall, these combined neural activities can be interpreted as local neural tools serving as prerequisites for task planning and other higher-order cognitive functions.

### What is the added value of the lateral prefrontal cortex in the working memory network?

The working memory network depends on a large set of cortical and subcortical nodes. ‘Delay neurons’ in the monkey, as well as activation in functional neuroimaging studies in both monkeys and humans, can be found in the lateral prefrontal cortex but also in the premotor, posterior parietal or inferior temporal cortices, as well as in different nuclei of the basal ganglia.^144,188-190^ It has been suggested that the parietal-premotor network is sufficient to maintain visuospatial information in working memory.^191-194^ However, as we have seen, activities related to the maintenance of the memory trace have been reported in a proportion of neurons in the lateral prefrontal cortex.^171^ In human neuroimaging studies, a classic finding is the step-like activation of the dorsolateral prefrontal cortex as a function of the amount of information contained in the working memory (‘the memory load’).^191,195^ However, this may reflect the involvement of executive processes such as those required for updating and monitoring information in the working memory and for managing proactive interferences as suggested by different studies in both humans and rhesus monkeys.^196-200^

In the rhesus monkey, several electrophysiological studies showed that during the delay period, a significant proportion of prefrontal neurons, rather than being involved in maintaining the sensory stimulus, are oriented towards preparing future actions.^171,174,201-205^ Similarly in humans, several functional neuroimaging and lesion studies indicate an essential role of the dorsolateral prefrontal cortex in selecting and preparing the response that is to be implemented rather than in maintenance processing.^104,194,206-212^ Taken together, these data suggest that, while the lateral prefrontal cortex can temporarily maintain a past experience, its activity responds primarily to the need to implement strategies to perform a goal-directed action.

### Can a rhesus monkey plan a series of actions?

Is a rhesus monkey capable of combining all these elementary operations to perform complex planning tasks similar to those that a human can perform? First, planning capacities are clearly limited by the fact that, in non-human primates, the working memory span is much lower than in humans. Although great apes can plan future possible events,^213^ a recent review of the literature on chimpanzee cognition underlines that the visual working memory span is 2 ± 1 items—no more than that of a 5-year-old human child—and it is associated with a low-level of performance in cognitive tasks relying on working memory, while in normal human adults, the span is 7 ± 1 items.^8^ Second, in accordance with this idea, rhesus monkeys can learn the ‘Self-Ordered’ task,^214,215^ similar to the human Self-Ordered task. This is a planning task that activates the lateral prefrontal cortex and is sensitive to lateral prefrontal damage in humans (Fig. 4B).^108^ However, it takes a particularly long time for rhesus monkeys to learn and stabilize the rule of a three-move plan of action (simpler than a five-move trial in the Tower of London task), with more than 3 months of daily learning required to achieve a 90% trial success rate in trials. A long learning phase was also observed in a single-cell recording study using a spatial sequencing task with three fixed targets, very similar to the Self-Ordered tasks.^173^ Taken together, these findings indicate that non-human primates can learn planning tasks close to those of a human but at the cost of considerable effort and with limited capacity.

In contrast, instead of studying the neural basis of macro-functions such as planning or problem-solving in humans, using tasks directly inspired by those designed for rhesus monkeys, ongoing neuroimaging and lesion studies in humans have demonstrated the critical role of lateral prefrontal subregions homologous to those of the rhesus monkey in working memory.^83,104,191,216-224^ This indicates that the human lateral prefrontal cortex processes the elementary functions (encompassed in working memory) required for planning or problem-solving.

### The primate ventrolateral prefrontal cortex: from response inhibition to categorization

The lateral prefrontal cortex also includes the ventrolateral region, which corresponds mainly, but not only, to the inferior frontal gyrus in humans (BA 44/45 and 47) and which, in the dominant hemisphere, includes Broca’s area. Anatomically, most of the ventrolateral human prefrontal cortex has a counterpart in the rhesus monkey.^148,225,226^ Like the dorsolateral region, the ventrolateral prefrontal cortex also contributes to cognitive control (e.g. decision-making, rule-learning and set-shifting).^227,228^ What is/are the basic, crucial elementary function(s) that depend(s) on the integrity of the ventrolateral prefrontal cortex and are common to human and non-human primates?

Several findings argue that a crucial role of the right posterior ventrolateral cortex (BA 44 in humans) is in response inhibition situations such as in the ‘Go/No-Go’ task [i.e. ‘when I tap once, tap once’ (go signal) ‘but if I tap twice, don't respond’ (no-go signal)]; the interference condition of the ‘Stroop’ task (i.e. naming the colour of the ink instead of the incongruent colour words; saying ‘red’ when the word ‘blue’ is printed in red); the ‘Anti-saccade’ task (i.e. making a saccade in the direction away from a stimulus); and the ‘Stop Signals’ task (i.e. responding as quickly as possible to a go cue but aborting any response when a stop cue is presented after the go cue). For instance, in humans, damage to the posterior right ventrolateral prefrontal cortex disrupts one’s ability to inhibit actions related to a relevant implicit or explicit stop signal through prefrontal-basal ganglia loops.^229-231^ Neuroimaging studies have implicated a consistent network of mainly right-lateralized brain regions encompassing frontal structures such as the dorsolateral and posterior ventrolateral prefrontal cortices, the anterior cingulate cortex and the pre-supplementary motor area, as well as bilateral parietal regions.^232-239^ Response inhibition can be fractionated into distinct subprocesses (i.e. interference control, action withholding and action cancellation) supported by distinct brain regions.^235,238^ Interference control involves processing information that is relevant to the task, while ignoring irrelevant information, and is mainly assessed by the Stroop task. Action withholding and action cancellation (the suppression of programmed but not yet initiated actions or already initiated actions)^235^ are tested mainly by the Go/No-Go and Stop Signal tasks, respectively. Although these processes appear to share common neural correlates, within the frontal lobes, action withholding and action cancellation might rely more specifically on the right posterior ventrolateral prefrontal cortex,^234,238,239^ which has been conceived as a brake on behavioural response.^231^ Finally, in the rhesus monkey, the involvement of the posterior ventrolateral prefrontal cortex is supported only by a single electrophysiological study,^240^ although the specific role of this area for this fundamental function has been little tested.^231^

Conditional motor learning or arbitrary visuomotor mapping (i.e. the arbitrary association between stimuli and actions such as a red light on the road indicating the driver to stop) is a fundamental function required for rule learning, decision-making, mental flexibility and, more generally, for cognitive control and goal-directed behaviour, depending on the context.^241^ It relies on a large distributed network in which the critical nodes are the lateral premotor cortex, the medial temporal lobes, the basal ganglia and the ventrolateral prefrontal cortex.^227,241,242^ Each node in this network may play a particular role at any given step of the mapping process.^241,243^ Based on lesion and electrophysiology studies in the rhesus monkey and on neuroimaging studies in humans, the ventrolateral prefrontal cortex seems to be essential to learning and consolidating the rule of association between the stimulus and the contextual actions, regardless of any working memory demand.^227,243^

The ventrolateral prefrontal cortex, thanks to its multimodal sensory integration capabilities,^227^ can combine object characteristics to find similarities between concrete objects in the external world, leading to their categorization.^107,244^ Categorization, the mental operation by which the brain classifies objects and events, is important in many domains of cognition and behaviour, from learning (e.g. children learn new concepts by categorizing items that look similar or have similar properties) to survival (e.g. to recognize an animal as dangerous, primates need to categorize it as similar to a previously encountered dangerous animal). When asked ‘In what way are an orange and a banana alike?’, normal subjects point to similarities and categorize them in the taxonomic category of the two objects. At least two complementary processes intervene: first, the ability to detect similarities between objects, and second, abstraction.^107,244,245^ Both in humans and the rhesus monkey, the ventrolateral prefrontal cortex, in coordination with the inferior temporal cortex, seems to play a central role in the process of visual categorization. This has been demonstrated by several functional neuroimaging studies in healthy subjects as well as electrophysiological studies both in humans and non-human primates.^246-259^ A fundamental function that seems to be common in both non-human primates and humans is the ability to find similarities between objects based on their concrete sensory (visual) features. First, in the rhesus monkey, when recording neurons below the lower bank of the principal sulcus (which corresponds, by homology, to the human ventrolateral prefrontal cortex) in visual categorization tasks such as, for example, those in which the monkey has to decide which category (‘dog’ or ‘cat’) to classify a hybrid animal containing varying amounts of dog and cat traits, one can observe neuronal activities related to taxonomic categorization based on visual features.^260-263^ This ability to categorize visual objects can be understood as being conveyed by essential connectivity routes in non-spatial cognition. The ventrolateral prefrontal cortex receives direct inputs via the uncinate fasciculus from the inferior temporal cortex, a central brain region for visual discrimination.^226,227,264,265^ Second, in humans, neuroimaging data, both in healthy subjects and in patients with diseases such as the behavioural variant of frontotemporal lobar degeneration, highlight the critical role of right anterior ventrolateral regions (BA 47) in identifying similarities between concrete visual objects (while the same region in the left hemisphere performs the same type of processing for verbal material).^107,244^ Overall, these results indicate that the ventrolateral prefrontal cortex of humans and rhesus monkeys is therefore a central region for a fundamental pillar of categorization, i.e. the ability to detect physical similarities between concrete objects. The fact that important categorization capacities exist in rhesus monkeys paves the way for more abstract categorization rules in humans, in line with what non-human primates can do (see discussion on abstraction later).

An important question concerns the specificity of Broca’s area and the surrounding regions in human language production. Can we find functions in the posterior ventrolateral prefrontal cortex of the rhesus monkey that could be the bases for language production in humans? First, there is a strong anatomical homology between humans, great apes and the rhesus monkey in the region that encompasses Broca’s area (BA 44/45 in the inferior frontal gyrus in humans).^225,266^ Second, as emphasized by Neubert *et al.*,^225^ Broca’s area does not only and specifically deal with language functions but also with processes that might represent pre-existing mechanisms for the emergence of human language such as arbitrary visuomotor mapping, multimodal integration, motor sequencing or imitation.^41,225,267,268^ However, although the whole explanation has not emerged fully, there are important differences regarding the posterior ventrolateral prefrontal cortex that may explain the absence of human-like language production in non-human primates: (i) a recent study has shown that a major structural difference lies in the lower volume of the neuropil in Broca’s area in non-human primates, indicating a lower connectivity that limits cognitive abilities^266^; (ii) consistent with lower connectivity, although in the rhesus monkey the posterior ventrolateral prefrontal cortex receives information from the auditory cortices,^269^ human posterior auditory association areas are more strongly connected with the ventrolateral prefrontal cortex than in other primates,^225^ which may explain why rhesus monkeys perform poorer on cognitive tasks based on auditory information than on visual ones^270^; and (iii) genetic differences such as the molecular structure of the gene coding for the *FOXP2* transcription factor, which is involved in vocal learning, and mutations in which can lead to disorders in the coordination of the orofacial movements required for speech, may be at the root of the limited development of the physiological factors required for language production.^271^

Finally, we must be careful not to create an artificial dichotomy between the elementary functions of the ventrolateral and dorsolateral regions. Numerous studies in rhesus monkeys and neuroimaging findings in humans have shown that the anterior ventrolateral prefrontal cortex (BA 47 in humans and Walker’s 12/47 in rhesus monkey) is involved in working memory functions.^196,216,221,222,272-277^ However, its specific contribution remains a matter of debate. Inversely, during the delay period of Delayed Response tasks, activation related to response inhibition can be found in the dorsolateral prefrontal cortex.^171^ Furthermore, a recent meta-analysis based on 68 functional neuroimaging studies (and 1684 participants) in humans showed that, in classical response inhibition tasks (Go/No-Go and Stop-Signal tasks), activation of the dorsolateral prefrontal cortex was constant and even higher than in the ventrolateral cortex for the greater effort to inhibit action.^278^

Overall, these data suggest that, for at least some of the functions performed by the lateral prefrontal cortex, there is a continuum rather than a gap between non-human primates and humans. This continuum encompasses several elementary critical functions such as working memory or response inhibition, although the functional and anatomical organization of the human lateral prefrontal cortex may differ from that of other primates.

However, it is obvious that despite this ‘cognitive’ continuity between non-human primates and humans, our societies and civilizations are quite different. Is it only a quantitative difference that turns into a qualitative leap in humans, or are there human functions that are absent or very poorly developed in non-human primates?

## Functions that are barely present or totally absent in non-human primates

### Differences in anatomical and functional organization between rhesus monkeys and humans

**Figure 5:**
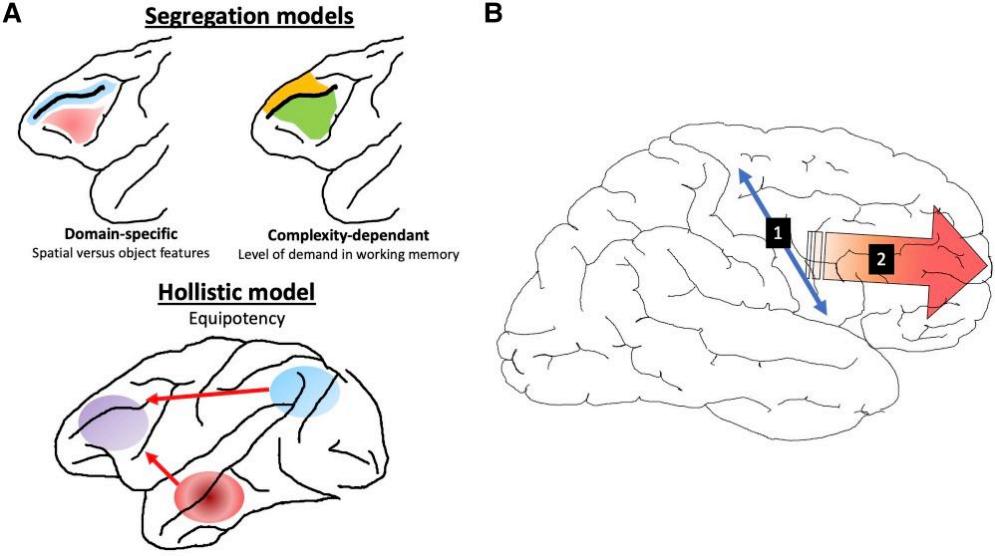
**Models of anatomical and functional organization of the prefrontal cortex in the rhesus monkey and humans**. (**A**) Anatomical and functional organization (AFO) models of the rhesus monkey. The blue-shaded area indicates the involvement of a given region in spatial cognition; the red-shaded area, an involvement in non-spatial (i.e. object) cognition; the purple-shaded area, a cross- or supra-model involvement; the green-shaded area, an involvement of the area under low working memory demand (e.g. simple maintenance); and the orange-shaded area, under higher working memory demand (e.g. updating information in working memory). (**B**) The current conception of prefrontal cortex AFO in humans, which comprises two orthogonal dimensions (or gradients). In the posterior lateral prefrontal cortex, one can observe a dorsal-ventral dimension (1) based on the domain of the information being processed (a verbal region in the inferior frontal gyrus, at least in the dominant hemisphere, and a spatial region in the superior frontal gyrus). According to Volle *et al.*,^87^ the intermediate region (the middle frontal gyrus) is cross- or supra-modal. The second dimension (2) is a caudal-rostral gradient. Two different but interrelated ideas coexist: this gradient is either based on the level of abstraction^47^ or on a combination of the load of information and the time/distance between the stimulus and the response.^19^

The most documented model of the anatomical and functional organization of the lateral prefrontal cortex in primates is the domain-specific model, which postulates that the lateral prefrontal cortex is divided into different subregions according to the domain of information being processed in the working memory.^26^ The processing of the spatial position of a visual stimulus, whatever its physical characteristics, is associated with the dorsolateral region (Walker’s areas 9 and 46), whereas the processing in working memory of the physical characteristics of objects and faces independently of their spatial location is associated with the ventrolateral region (Walker’s areas 12 and 45).^26,272,273,279^ However, the domain-specific model has been challenged by two other models (Fig. 5A). The first of these also segregates the lateral prefrontal cortex but based on the nature of the operations performed in working memory (the dorsolateral region is involved in the manipulation and sequential management of information held in working memory, while the ventrolateral region is thought to be involved in maintaining working memory and performing behaviours that require little or no mental manipulation).^44,83,214^ The second of these, a holistic model, argues in favour of a relative equipotency of neuron functions, given that the prefrontal cortex is at the heart of fluid intelligence and mental flexibility and that, consequently, the lateral prefrontal cortex is not divisible into several subregions that differently process information stored in working memory. Instead, this mental resource management function requires the integration of multimodal sensory information,^14^ and neuronal tuning may change according to the context of any given task.^179,274,275,280^ Such a debate has also been transferred to the human lateral prefrontal cortex.^44,87,180,220,221,277,281^

So far, none of the models proposed in rhesus monkeys has been fully replicated in humans. On the contrary, a very different picture seems to emerge. First, Volle *et al.*^87^ used a multimodal approach, combining cognitive tasks that addressed the different models in the monkey. The study used the classical ‘n-back’ working memory tasks, in which one should tell whether the current stimulus matches that from one, two or three steps earlier (the 1-, 2- or 3-back tasks). It is possible to cross-reference the level of updating (1-, 2- or 3-back) in working memory with the domain of information (e.g. spatial locations, faces or letters) using fMRI in healthy subjects and patients with post-stroke focal brain lesions, as well as using voxel-based lesion-symptom mapping, enabling detection of the voxel clusters most critical for a given behaviour in patients with focal brain lesions.^282-284^ With this multimodal approach, it has been shown that none of the available anatomical and functional organization models in monkey can account for what was found in humans. First, only the highest demand in executive control (3-back tasks) seems to involve the lateral prefrontal cortex. Second, only the most posterior part of the lateral prefrontal cortex seems to participate critically in the performance of these tasks. Third, within the posterior lateral prefrontal cortex, one can find both domain-dependent subregions (spatial and verbal) as well as subregions that are either cross- or supra-modal.^87,105^ In many respects, these data are consistent with those obtained by other studies.^192,196,277,285,286^ Furthermore, based on functional imaging and tractography data, Thiebaut de Schotten *et al.*^40^ showed that the lateral surface of the prefrontal cortex can be subdivided into many subregions from a combined network connectivity and functional approach.

### The emergence of caudal-rostral models

Consistent data from different teams have shown that the model that seems to prevail in humans is based on a caudo-rostral functional gradient, relying on an increase in the quantity of information to be processed, an increase in the temporal distance between the information and the response to be made, and an increase in the level of abstraction.^19,20,46,47,285,287-291^ In other words, ‘the more rostral, the more complex’ (Fig. 5B).

More precisely, two different but overlapping models can be discussed. Koechlin *et al*.^19,46,288^ proposed a model of cognitive control organized along the caudo-rostral axis, according to which, the more anterior in the prefrontal cortex, the greater the amount of information processed and the longer the interval between stimulus and response. Three different regions along the caudo-rostral axis emerged: (i) the posterior frontal cortex (BA 6/8), involved in what was named ‘sensorimotor control’—an expected event leads to an immediate response (someone rings at your door; you open it); (ii) the lateral regions (BA 44/45), involved in ‘contextual control’—appropriate decoding of the current context will trigger the accurate action (you are at your friend’s home and someone rings at the door; you won’t open the door and will let your friend do so); and (iii) the dorsolateral regions (BA 9/46), involved in ‘episodic control’—a specific past experience or information will provide the essential clue for triggering the expected action (you are at your friend’s home; your friend is not there, but before leaving, your friend tells you that if someone rings you should open the door). It is interesting to note that this model joins other models by taking into account fundamental operations previously attributed to each of the regions described in this model. For example, sensorimotor control, involving lateral premotor regions (BA 6/8), corresponds to arbitrary visuomotor mapping, associated with the lateral premotor cortex in both monkey and human studies.^241,242,292,293^ Contextual control, related to areas BA 44/45, requires slowing down, ‘braking’ immediate action as well as learning a new stimulus-response association, thereby matching the findings of studies regarding the role of the ventrolateral prefrontal cortex in both inhibition and arbitrary visuomotor mapping. Eventually, episodic control, involving areas BA 9/46, triggers executive aspects of working memory and inhibition and may explain the mid-dorsolateral prefrontal focus. This model is very similar to a second one based on the same division of the lateral regions of the prefrontal cortex but differing in the nature of the operations implemented, this time based on the increasing level of abstraction from the caudal to the rostral prefrontal cortex.^20,47,287,289^

### The role of the lateral frontopolar cortex in humans

Comparative anatomy between humans and non-human primates (rhesus monkeys and chimpanzees) shows that few prefrontal regions are non-homologous in humans. Those that do not seem to have a counterpart in humans are the rostral prefrontal regions, which could be one of the central elements distinguishing human cognition from that of other primates.^43,148^ The question is then, what could be the functions of these partially new brain regions in humans?

On one hand, the model developed by Koechlin *et al.*^19,46,288^ can be supplemented by an additional component, ‘branching’, activating the most anterior regions of the prefrontal cortex (lateral rostral prefrontal cortex/BA10).^294^ In this case, the additional function consists of the ability to interrupt a complex task (such as updating information in working memory) in the middle of said task, switch to an interfering task, and then resume the first task at the point where it was left off. It can therefore be suggested that one of the essential roles of the lateral rostral prefrontal cortex is to merge temporally distant knowledge to expand executive control. On the other hand, in the model of abstraction developed by Badre and colleagues,^20,47,287,289^ the more anterior, the less concrete the information, the more rules are processed and the more temporally distant the information, allowing for long-term goals. This led Badre and Nee^289^ to propose that this model of anatomical and functional organization manages ‘temporal abstraction’. Therefore, both models agree that one of the functions of the most anterior regions is to temporally link information that is distant from each other.

In addition, the particular development of the rostral lateral prefrontal cortex in humans seems to be associated with our abilities for analogical reasoning and creative thinking.^85,86,295,296^ Reasoning by analogy depends on the ability to consider, integrate and compare multiple relationships between components of mental representations.^297-299^ Along the same lines, among the processes involved in creativity (defined as the ability to produce work that is both novel/original and appropriate or useful^300-302^) and within the large distributed brain network that contributes to creative thinking,^295,303^ the ability to link distant semantic knowledge and think away from pre-established associations seems to be associated with the rostral lateral prefrontal cortex.^295,304,305^ For instance, selective rostral prefrontal activation is found in divergent thinking tasks such as the ‘Alternate Use’ task, in which one should find the alternative use of a given object (e.g. ‘pen’), or in associative combination tasks such as the ‘Remote Associates Test’, in which a target word (e.g. ‘memory’) should be guessed when three other words are proposed (‘elephant’, ‘lapse’, ‘vivid’). In both cases, participants should move away from the most obvious association and create/activate links between more distant semantic knowledge. Together, this set of data suggests that one essential role of the rostral lateral prefrontal cortex is to link distant semantic information. Combined with the two above-described rostro-caudal models of the anatomical and functional organization of the prefrontal cortex, these data support the general idea that one of the essential roles of the lateral rostral prefrontal cortex is to merge temporally or semantically distant knowledge to expand executive control. The idea that the frontopolar region is essential for linking distant knowledge can also explain the involvement of this area in implementing task rule, which requires assembling various elements, often arbitrarily linked, to form the rule to be applied.^291,306,307^

## General summary

More than five decades of research have shown that goal-directed behaviours are based on macro-functions such as, among many others, decision-making, planning or reasoning, which in turn rely on more elementary processes common to all primates (e.g. working memory, response inhibition, arbitrary visuomotor mapping, similarity findings). These findings suggest that there is a continuum rather than a gap in prefrontal functions between non-human primates and humans.

That said, it nevertheless appears that despite this continuity, cognitive abilities and achievements (the degree of civilization) are strikingly different between humans and non-human primates. First, although there is functional continuity, basic processes such as working memory are much more highly developed in humans than in monkeys. Second, working memory operations, which appear to occupy most of the lateral prefrontal cortex in rhesus monkeys, are shifted to the posterior portion of the lateral prefrontal cortex in humans, paving the way for the emergence of other, more developed, cognitive functions. Third, the spectacular development of the frontopolar cortex (BA 10) appears to be accompanied by the ability to process information in a more abstract manner (allowing links to be made between physically distant but semantically related information) and to extend the temporal space of representation by creating links between temporally distant information.

Therefore, we propose that three major cognitive changes differentiate humans from other primates: (i) the expansion of the mental space of representation in working memory, allowing greater integration of past experiences and prospective futures; (ii) interrelated to the first point, and largely due to the development of the lateral frontopolar cortex, a greater capacity to link discontinuous or distant data, whether temporal or semantic; and (iii) a greater capacity for abstraction, allowing us, beyond the concrete data immediately accessible to our perception, to classify knowledge in different ways (taxonomic classification), to engage in analogical reasoning or to acquire abstract values that give rise to our beliefs and morals.

## Hypotheses and speculations regarding cognitive changes over the course of human evolution

These three major cognitive changes occurred during, and probably in concert with, the evolution of our species. Indeed, lithic technology (the manufacture of the first tools, about 2 to 3 million years ago) indicates that the creation of tools is associated with an understanding of the purpose for their use. Furthermore, the making of tools to create other tools indicates that the capability to represent abstract thinking in a mental space of representation and link temporally distant information was already well developed. Later, the manufacture of the first huts, 400 000 years ago, testified to the ability to draw up construction plans (i.e. the ability to represent a future and complex object that does not exist in the present). The first symbolic representations of the world such as the chimerical drawings (i.e. mixing features of different animals to produce a non-existing creature) painted in caves and the first sculptures (20 to 40 000 Bc) testified to an even higher level of abstraction. One of the interesting ideas regarding the evolution of prefrontal functions in *Homo* species has recently been proposed by Read *et al.*^8^ It is assumed that the common ancestor to all Hominidae (humans and great apes) had a working memory span equal to that of chimpanzee (2 ± 1), and then in *Homo* species it increased linearly to 7 ± 2, with an initial ‘jump’ from 2 to 3 that made a definitive cognitive difference. This assumption compares with archaeological evidence for qualitative changes marking different stages in the design and technological complexity of tool manufacture.

Language, defined as communication based on symbolic representations, appeared very late in the evolution of our species. Indeed, the first traces of symbolic communication were observed in the use of tokens as currency, 9000 Bc, and then formally proven by cuneiform writing, 3500 Bc.^308-312^ Communication based on symbolic representations (language) is only possible if the capacities for abstraction and working memory are particularly well developed. This indicates that the emergence of language depends to a large extent on the prior development of the three cognitive changes characteristic of the human prefrontal cortex. However, it is difficult to reduce human language to abstraction, distant semantic links and working memory. Indeed, for many linguists, the main characteristic of human language is its recursive and combinatorial syntax, although no consensus has been reached on this issue.^313-315^ However, combinatorial thinking also depends to a large extent on frontal functions and in particular on the ability to manipulate mental representations in working memory^26^ and to combine multiple rules.^294,316^

From the idea that language is in part derived from the progressive increase of abstraction, we would like to make the assumption that each aspect of the most elaborate human behaviour is associated with the three cognitive changes described above. This is the case for those thoughts or behaviours that are specifically human and rely strongly on abstract thinking such as our moral beliefs and values or our complex social relationships, which are based on a cognitive construction of empathy and a high level of inference about other people’s thoughts.^312,317-322^ Generally speaking, by enriching this mental space for deliberation and decoupling the immediate perception from the forthcoming action, we have become capable of creating additional degrees of freedom from our environment and our archaic, impulsive behaviour, conferring a source of imagination to represent alternative or new options (creativity), and therefore, what philosophers name, ‘free will’.

## Towards a new model unifying non-human primates and humans

The functions of the prefrontal cortex can be understood only in terms of its anatomical relationships and therefore its connectivity,^26,40,227^ in particular with the posterior associative regions that supply it with sensory information that is essential for establishing context and making decisions. Accordingly, and on the basis of a wide range of scientific data, two recent theories proposed by Genovesio *et al*.^24^ about the dorsolateral prefrontal cortex and Eldridge *et al*.^25^ about the ventrolateral prefrontal cortex can be unified to shed light on the general principles of the functions of the prefrontal cortex (lateral, at least) in a connectionist vision that also takes into account the evolution of primates and the different use of cognitive resources depending on the species.

The dorsolateral and ventrolateral regions receive their inputs from different posterior associative cortices: the dorsolateral region mainly from the posterior parietal cortex, forming the frontoparietal executive network (or central executive network); and the ventrolateral cortex from the inferior temporal cortex, via the uncinate fasciculus.^264,323^ If, instead of focusing on the prefrontal cortex, we choose the frontoparietal and frontotemporal networks as the unit of observation, they each form functional entities that process, integrate, contextualize or use very different types of information. The posterior parietal cortex will integrate and transmit to the dorsolateral prefrontal cortex information concerning temporal order, the number of items, duration, length, distance and proportion, whereas the inferotemporal cortex integrates and transmits to the ventrolateral prefrontal cortex information concerning the particular features of an object, such as visual texture, curvature or glossiness and their conjunction.^24,25^

In both cases, the information transmitted by these posterior cortices to the different regions of the lateral prefrontal cortex is used to achieve goal-directed behaviour. While it is clear that humans and non-human primates behave in a goal-directed way to maintain survival, the objects they seek for survival are not the same. For our ancestors and modern primates, foraging for food was/is one of the key elements of survival. The quest is concrete and based on sensory data that are immediately accessible or have a limited level of abstraction (such as ‘greater’, nearer, ‘better’).^24^ For us humans, the goals that seem essential are often much more abstract, less related to the immediate present and sometimes based on values counterintuitive to survival, like sacrificing ourselves to save the lives of others, knowing full well that in doing so we will die. Explicitly or implicitly, Genovesio *et al.*^24^ as well as Eldridge *et al.*^25^ suggest a transition from the foraging behaviour of non-human primates to foresight in humans, using the same brain networks.

Here, we hypothesize that the involvement of the lateral prefrontal cortex in the transition from foraging to foresight is made possible by the anatomical changes and three cognitive changes described earlier in this review. In humans in particular, the increase in connectivity and the appearance of new anatomical areas that are not completely homologous to those in monkeys, give rise, among other things, to new capacities for abstraction or language, allowing goals to be achieved that are different from those of other primates. These new abilities can therefore be seen as an extension of, rather than a departure from, those of other primates.

## Conclusion: the giant panda enters the debate

The question, which we cannot yet fully answer with certainty, is to understand why in primates and not in other mammals, and then why in humans and not in other primates, the prefrontal cortex has evolved to develop in such a way. This question would warrant a very long essay and is one the author is not competent to answer, although available work has proposed convincing theories such as the particular development of social interactions in Homindae or the impact of climate change, from humid to dry, favouring a major evolutionary change based on bipedalism, freeing upper limbs and hands to handle objects like stone.

However, by analogy, we can apply to humans what paleontologist Stephen Jay Gould proposed about the giant panda to explain how environmental pressure led to a crucial adaptive change for the animal.^324^ In short, the giant panda is an Ursidae. Bears have five fingers and a small lateral ‘hand’ bone, called the sesamoid radial bone, which is particularly developed. However, this excrescence does not give the bear any adaptive advantage. In the giant panda, the excrescence of the sesamoid radial bone has given birth to a sixth finger—an opposable thumb—allowing the panda to grasp bamboo stalks firmly, bamboo being its only source of food. In conclusion, it is very likely that under environmental pressures that challenge survival (such as those our ancestors witnessed), a pre-existing organ that did not confer any decisive advantage turns into a highly adaptive structure. Could this idea be applied to the prefrontal cortex in primates and particularly in human kind?

## Glossary

**Delay neurons:** Neurons for which activity is maintained during the performance of tasks in which a delay period separates the cue (no longer visible) from the action (that should be held back).

**Dorsolateral prefrontal cortex:** The upper half of the lateral prefrontal cortex. In humans, this is represented by the superior frontal gyrus and the upper part of the middle frontal gyri. It corresponds to Brodmann areas 9 and 46 (upper part).

**Frontopolar cortex:** The most anterior (rostral) part of the prefrontal cortex. It corresponds to Brodmann area 10.

**Dysexecutive syndrome:** A clinical syndrome in which the capacity to plan and carry-out complex goal-directed behaviour is impaired.

**Granular cortex:** A six-layer cortex in which the fourth layer, composed of granular cells, is particularly developed. It is specific to primates.

**Great apes:** Chimpanzee, pygmee chimpanzee (bonobo), gorilla and orangutan.

**Hodology:** The study of pathways.

**Homology:** A homologue expresses a correspondence between one set and another (different from analogy and identity); here, between a given prefrontal region in different species of primates.

**Hominidae**: Humans and great apes.

**Macro versus elementary/fundamental functions:** Macro-function is a mental function such as planning that is based on several more elementary operations combined. An elementary/fundamental function here is a function on which more global (macro-) functions are based. An elementary/fundamental function is necessary but insufficient and needs to be combined with other elementary functions if the macro-function is to be realized.

**Old-world versus new-world monkeys:** Here, old-world refers to Africa and Asia, new world to central and south America. Greats apes, rhesus monkeys and baboons are old-world monkeys. Capuchins, *Saimiris* and *Ateles*, to name a few, are new-world monkeys.

**Principal sulcus (sulcus principalis):** The sulcus that separates the lateral surface of the prefrontal cortex at its middle in the rhesus monkey.

**Ventrolateral prefrontal cortex:** The lower half of the lateral prefrontal cortex. In humans, it is represented by the inferior frontal gyrus and the lower part of the middle frontal gyrus. It corresponds to Brodmann areas 44, 45, 46 (lower part) and 47.

**Walker’s areas**: One of the classic classifications of cytoarchitectonic areas in the rhesus monkey (frequently used in the literature, until recent works that have revisited the anatomy of the prefrontal cortex).

**Working memory:** A set of mental processes allowing maintenance and manipulation of mental representation in order to provide adapted forthcoming actions.

**Working memory span:** The quantity of individual items that can be maintained in working memory.
